# Laryngeal mask airway reduces incidence of post-operative sore throat after thyroid surgery compared with endotracheal tube: a single-blinded randomized controlled trial

**DOI:** 10.1186/s12871-020-0932-2

**Published:** 2020-01-14

**Authors:** Yahong Gong, Xiaohan Xu, Jin Wang, Lu Che, Weijia Wang, Jie Yi

**Affiliations:** Department of Anesthesiology, Peking Union Medical College Hospital (PUMCH), 1 Shuai Fuyuan, Wangfujing Street, Dongcheng District, Beijing, 100730 China

**Keywords:** Sore throat, Thyroid surgery, Postoperative, Endotracheal tube, Flexible laryngeal mask airway

## Abstract

**Background:**

Sore throat is a remarkable complication after thyroid surgery with endotracheal tube (ETT). Many studies revealed that laryngeal mask airway (LMA) might reduce the incidence and severity of postoperative sore throat. However, little is known about the use of a flexible reinforced LMA (FLMA) in thyroid surgery. The purpose of this study was to explore the potential benefits of FLMA compared with ETT on postoperative sore throat.

**Methods:**

In this prospective, single-blinded, randomized, controlled trial, ninety-six patients aged 20–80 years, scheduled for elective radical thyroidectomy under general anesthesia were enrolled. They were randomly divided into ETT group and FLMA group. All the included patients received total intravenous anesthesia (with propofol, fentanyl and rocuronium) and controlled mechanical ventilation during the surgery. Cuff pressure of ETT and FLMA were strictly controlled. Incidence and severity of postoperative sore throat, numbness and hoarseness at 1, 24, and 48 h after surgery was evaluated and compared between the two groups. Incidence and severity of buckling during extubation and the hemodynamic profile during intubation were also recorded and compared.

**Results:**

The incidence of sore throat and hoarseness was significantly lower in FLMA group than those in ETT group at 1 h, 24 h and 48 h postoperatively, as well as the severity of sore throat. Compared to ETT group, there was a significantly lower incidence of buckling during extubation and less fluctuation of HR and BP at 1 min and 3 min after intubation in FLMA group.

**Conclusions:**

Patients undergoing thyroid surgery with FLMA had less postoperative laryngopharyngeal symptoms when compared with ETT. The use of FLMA also achieved less buckling during extubation and better hemodynamic profiles during intubation.

**Trial registration:**

The research was registered in Chinese Clinical Trial Registry (ChiCTR-IOR-15006602) on May 23th, 2015.

## Background

Sore throat is a common complication after general anesthesia with endotracheal tube (ETT) [1]. Incidence of moderate to severe sore throat varies from 15 to 62% [1–5]. In the case of thyroid surgery, an even higher incidence of approximately 68.4% was reported [6–8]. Postoperative sore throat could last 2 to 3 days, and could be a real concern for patients and causes distress and anxiety during the recovery [9], thus may need additional attention from doctors and nurses. This problem would be even more prominent in ambulatory and overnight stay thyroid surgery. Many studies suggested that laryngeal mask airway may reduce potential damage to the vocal cord and thus prevent postoperative laryngopharyngeal symptoms [10]. In 1991, Greatorex et al. firstly published their experience of using LMA in thyroid surgery [11]. However, little information has been reported about the use of a flexible reinforced LMA (FLMA) during thyroidectomy and the incidence of postoperative laryngopharyngeal discomfort. With the clinical goal of reducing the incidence of postoperative sore throat, the aim of this study was to explore the potential benefits of FLMA compared with ETT on postoperative sore throat.

## Methods

### Subjects

This single-blinded, parallel, controlled trial with equal randomization was approved by the Institutional Review Board of Peking Union Medical College Hospital (PUMCH) (IRB No. S-631) and registered in Chinese Clinical Trial Registry (ChiCTR-IOR-15006602) on May 23th, 2015. Informed consent was obtained from all individual participants included in the study. The study protocol adhered to CONSORT guidelines.

Nighty-six patients with American Society of Anesthesiology Physical Status Classification (ASA)I-II, aged 20–80 years, scheduled for elective radical thyroidectomy under general anesthesia from May 26th 2015 to April 2016 were enrolled. Exclusion criteria were: preoperative sore throat, hoarseness, dysphagia, high risk of regurgitation or aspiration, obesity (body mass index, BMI > 30 kg/m^2^), symptoms of upper respiratory infection within 2 weeks before surgery, previous surgeries of the oral cavity or pharynx, and estimated surgery time > 4 h. All the patients scheduled for thyroid surgery received laryngofiberscope preoperatively to evaluate the function of vocal cord. Cases without definitive identification of recurrent laryngeal nerve or with mechanical or thermal injury of recurrent laryngeal nerve during surgery were also excluded.

### Study design

The admitted patients were randomly assigned to ETT group (ETT group, *n* = 48) or flexible reinforced LMA group (LMA group, *n* = 48) by a computer-generated table of random numbers immediately prior to surgery. No restriction was used for randomization. The random numbers for assignment were sealed in opaque envelopes, which could only be opened by the anesthesia providers.

The study was conducted in the operating rooms in Peking Union Medical College Hospital in Beijing, China. In ETT group, patients were intubated with a high-volume, low-pressure-cuff plain endotracheal tube (Covidien, Mexico). In order to control the possible bias, smaller sized ETTs (size 7.0 for female patients and 7.5 for male) were chosen for our patients, which were reported to be associated with a lower incidence of sore throat [6]. The cuff of ETT was inflated with room air, and cuff pressure was strictly adjusted to 25cmH_2_O with a handheld aneroid manometer (VBM, Einsteinstr, Germany). In LMA group, a flexible reinforced LMA (LMA Flexible™, Laryngeal Mask Company Limited, Seychelles, Singapore) was used according to the patient’s body weight (BW) (size 3 (BW < 50 kg), 4 (BW 50–70 kg), or 5 (BW > 70 kg)). The cuff of flexible reinforced LMA was fully deflated before insertion. After lubrication of the posterior surface with water-based jelly, FLMA was inserted with digital intraoral manipulation. Cuff pressure of FLMA was adjusted to 40cmH_2_O with manometer. Proper position was confirmed by a visualization of more than half vocal cords through bronchoscopy.

The patients, data collectors and outcome assessors were blinded to the group assignment. In order to ensure patients’ safety, the anesthesiologist in charge of the anesthesia was not blinded to the group assignment. ETT and FLMA were placed by two experienced anesthesiologists who had successfully inserted ETT for over 300 times in thyroid surgeries and ETT for over 300 times in other surgeries. All the surgeries were carried out by one surgical team.

### Anesthesia management

On arrival at the operating room, all the patients received midazolam 0.03 mg·kg-1 as a premedication. Intraoperative monitoring included electrocardiography, noninvasive blood pressure, pulse oximetry (SpO_2_), capnograpy (EtCO_2_), gas analysis, tidal volume, airway pressure and bispectral index (BIS). Anesthesia was induced with target-controlled infusion of propofol (at effect-site concentration of 3–3.5 μg/ml) and bolus injection of fentanyl 2 μg/kg and rocuronium 0.6 mg/kg.

During the operation, anesthesia was maintained with intermittent bolus injection of fentanyl and target controlled infusions (TCI) of propofol using Graseby 3500 Anaesthesia Syringe Pump - Diprifusor (Smiths-medical, UK). The systolic blood pressure fluctuation was maintained within 15% of baseline and BIS value between 40 and 60 (Aspect XP, space Lab, USA). Propofol infusion was discontinued around 10 min before the end of surgery and neostigmine 6 mg was given to antagonize the residual muscle relaxant. FLMA/ETT was extracted when the patient could follow voice commands and EtCO_2_ was bellow 45 mmHg on spontaneous respiration. None of the patients received additional analgesics in PACU or wards during postoperative 48 h.

### Data collection

The primary end point was the incidence and severity of postoperative sore throat. Secondary end points were: (1) the incidence and severity of buckling during extubation, (2) postoperative numbness and hoarseness, (3) hemodynamic profiles. Laryngopharyngeal symptoms were evaluated at 1, 24, and 48 h after surgery by three anesthesiologists who were blinded to the treatment group of the patient. Sore throat was assessed using a visual analog scale (VAS, 0 = none, 10 = most severe). Numbness was evaluated according to patients’ self-report. Hoarseness was assessed based on whether there were changes of the voice to harsh or strained. The scale used to evaluate the severity of buckling during extubation was a three-graded scale: 0 = None, 1 = Mild buckling (once/twice cough without head lifting-off the bed and lasting less than 15 s), 2 = Severe buckling (cough more than twice with head lifting-off the bed or lasting longer than 15 s). In addition, the diastolic blood pressure (DBP), systolic blood pressure (SBP) and heart rate (HR) [12] were recorded the time before, at 1 min, 3 min and 5 min after endotracheal intubation/insertion of LMA.

### Statistical analysis

Previous study showed that the incidence of sore throat at 6 h after thyroid surgery was 84% for the control group [5]. If a 30% decrease in the incidence of sore throat was considered clinically significant, power analysis program (G* power 3.1) calculated that 35 patients per group were needed for a power of 80% and an error of 0.05. Considering a 10% dropout rate, 38 patients for each group were necessary. SPSS software (version 13.0; SPSS, Inc., Chicago, IL, USA) was used for data analysis. Continuous normally and non-normally distributed data was described as mean ± standard deviation and median [13], respectively. Between-group demographic data with normal distribution was analyzed with unpaired t-test or Chi Square test, if appropriate. Hemodynamic data at each time point was compared by one-way ANOVA. The incidences of sore throat, hoarseness, numbness and buckling were analyzed using Fisher’s exact test. The Mann–Whitney U test was used to compare the severity of sore throat. A two-sided *p*-value < 0.05 was considered statistically significant.

## Results

### Demographic characteristics

**Fig. 1:**
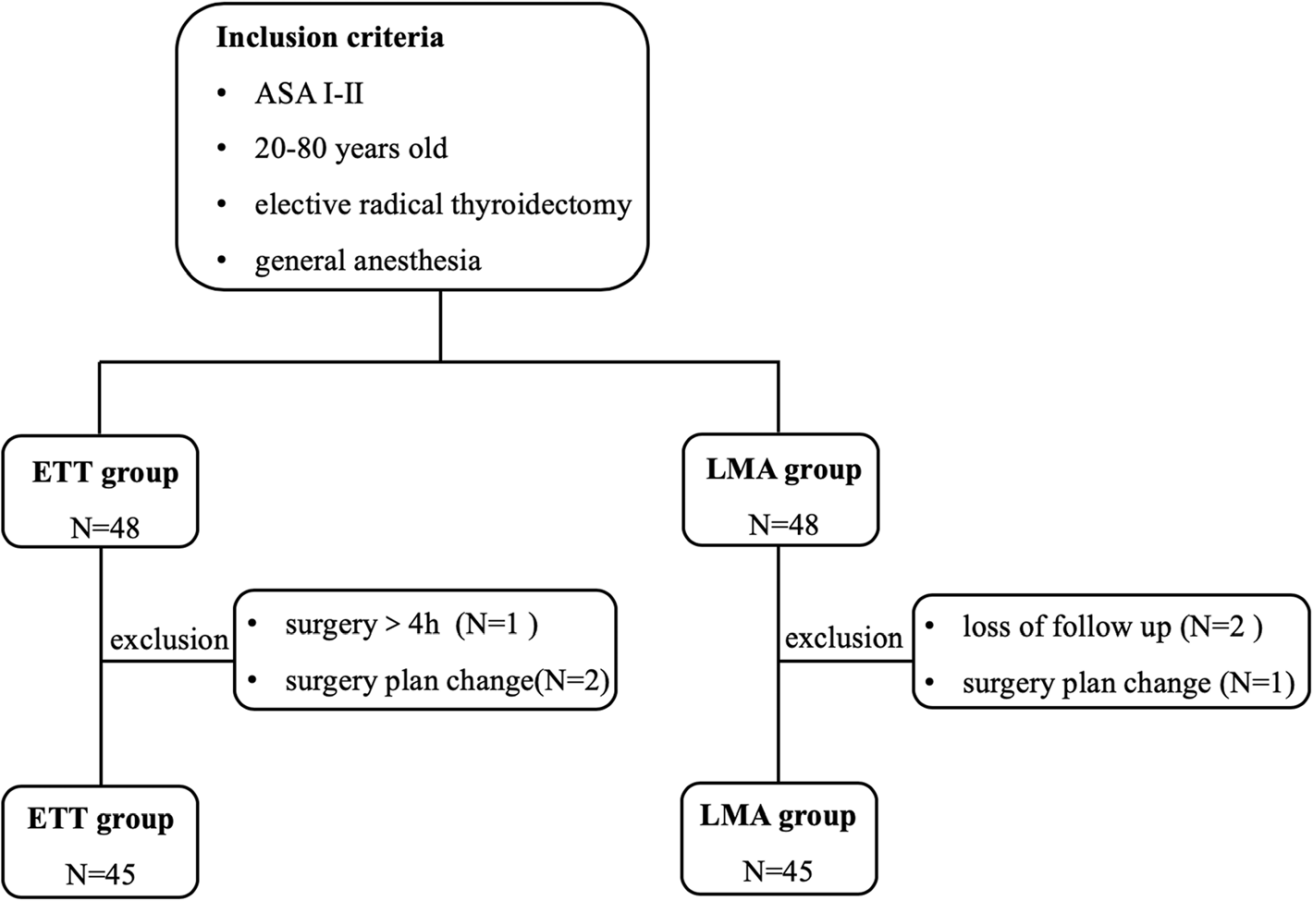
The inclusion and exclusion of patients. Abbreviations: ASA, American Society of Anesthesiology Physical Status Classification; LMA, laryngeal mask airway; ETT, endotracheal tube

A total of 90 patients from May 2015 to April 2016 were included in the final analysis. One patient from ETT group was excluded since surgery time lasted longer than 4 h. Three patients (2 from ETT group and 1 from LMA group) were excluded during the study period due to a change in the operation plan. Another two patients (from LMA group) were excluded because of loss of follow up (Fig. 1). Demographic characteristics are presented in Table 1. There were no differences between the groups regarding age, gender, body weight, height and duration of surgery.

**Table 1:** Demographic data

	Group ETT (*n* = 45)	Group LMA (*n* = 45)	*p*-value
Age (yrs)	42.1 ± 10.9	42.7 ± 10.6	0.777
Male/Female (n)	9/36	8/37	0.788
Body Weight (kg)	64.1 ± 10.2	65.5 ± 13.3	0.580
Body Height (cm)	164.7 ± 5.7	165.1 ± 7.3	0.711
Operative time (min)	48.20 ± 21.45	54.00 ± 21.63	0.205

Values are means ± SDs or number of patients. Between-group demographic data with normal distribution was analyzed with unpaired t-test or Chi Square test, if appropriate. A two-sided *p*-value < 0.05 was considered statistically significant

*Abbreviations*: *ETT* endotracheal tube, *FLMA* flexible laryngeal mask airway

### Postoperative laryngopharyngeal symptoms

The incidence of sore throat was significantly lower in group LMA than in the group ETT at 1 h (48.9% vs 68.9%, *p* < 0.001), 24 h (37.8% vs 51.1%, *p* = 0.012) and 48 h (6.7% vs 24.4%, *p* = 0.023) postoperatively (Table 2). The incidence of hoarseness was also significantly less in the group LMA than in the group ETT at 1, 24 and 48 h postoperatively (8.9% vs. 57.8%, *p* < 0.001; 6.7% vs. 28.9%, *p* < 0.001; 0% vs. 13.3%, *p* = 0.002) (Table 2). Postoperative numbness was comparable in two groups (Table 2). The severity of sore throat in the group LMA was significantly lower than in the group ETT at 1 h (0[0–4] vs. 2 [0–7], *p* = 0.006) and 48 h (0 [0–1] vs. 0 [0–2] at 48 h, *p* = 0.017) after surgery (Table 3). VAS score of sore throat was higher in the group ETT than in the group LMA at 24 h after surgery, but the difference was not significant.

**Table 2:** Postoperative laryngopharyngeal symptoms

	Group ETT (*n* = 45)	Group LMA (*n* = 45)	*p*-value
Sore throat			
PO 1 h	31 (68.9%)	22 (48.9%)	< 0.001*
PO 24 h	23 (51.1%)	17 (37.8%)	0.012*
PO 48 h	11 (24.4%)	3 (6.7%)	0.023*
Hoarseness			
PO 1 h	26 (57.8%)	4 (8.9%)	< 0.001*
PO 24 h	13 (28.9%)	3 (6.7%)	< 0.001*
PO 48 h	6 (13.3%)	0 (0%)	0.002*
Numbness			
PO 1 h	1 (2.2%)	3 (6.7%)	0.738
PO 24 h	1 (2.2%)	0 (0%)	0.085
PO 48 h	0 (0%)	0 (0%)	NA

Values are expressed as number of patients (percentage). The incidences of sore throat, hoarseness and numbness were analyzed using Fisher’s exact test. A two-sided *p*-value < 0.05 was considered statistically significant. * *p<0.005*

*Abbreviations*: *ETT* endotracheal tube, *FLMA* flexible laryngeal mask airway, *PO* post-operative, *NA* not applicable

**Table 3:** Severity of sore throat

	Group LMA (*n* = 45)	Group ETT (*n* = 45)	*p*-value
PO 1 h	0 (0–4)	2 (0–7)	0.006
PO 24 h	0 (0–3)	1 (0–5)	0.07
PO 48 h	0 (0–1)	0 (0–2)	0.017*

Values are presented as median (range). VAS, *0* none, *10* most severe. The Mann–Whitney U test was used to compare the severity of sore throat. A two-sided *p*-value < 0.05 was considered statistically significant. * *p<0.005*

*Abbreviations*: *ETT* endotracheal tube, *FLMA* flexible laryngeal mask airway, *PO* post-operative

### Hemodynamic profiles and buckling

**Fig. 2:**
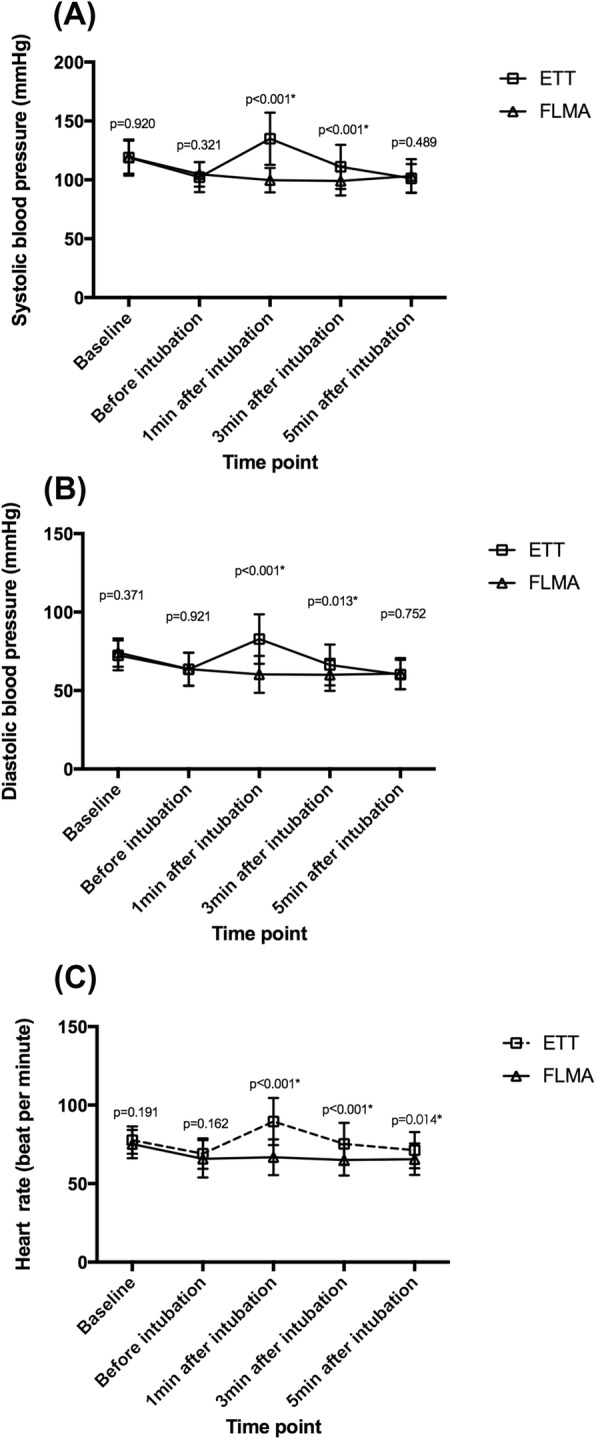
Hemodynamic Profiles and Buckling. **a** systolic blood pressure changes during intubation, **b** diastolic blood pressure changes during intubation, **c** heart rate changes during intubation. Data between FLMA and ETT group at each time point was compared by one-way ANOVA and a *p* < 0.05 was considered to be statistically significant. *p < 0.05. Abbreviations: FLMA, reinforced laryngeal mask airway; ETT, endotracheal tube

Baseline and pre-intubation HR, SBP and DBP were comparable between the two groups, but the values were significantly lower in Group LMA than in Group ETT at 1 min and 3 min after endotracheal intubation or insertion of FLMA (Fig. 2). There was a significantly lower incidence of buckling during extubation in the group LMA compared to the group ETT (*p* < 0.001, Table 4).

**Table 4:** Incidence of buckling during extubation

	Group LMA (*n* = 45)	Group ETT (*n* = 45)	*p*-value
Buckling			
0	43 (95.6%)	16 (35.6%)	< 0.001*
1	2 (4.4%)	17 (37.8%)	
2	0 (0.0%)	12 (26.7%)	

Values are presented as number (percentage). The incidences of buckling were analyzed using Fisher’s exact test. A two-sided *p*-value < 0.05 was considered statistically significant. * *p<0.005*

*Abbreviations*: *ETT* endotracheal tube, *FLMA* flexible laryngeal mask airway

## Discussion

The results of this study showed that compared with the endotracheal tube, the use of a flexible LMA during thyroidectomy decreased the incidence and severity of postoperative laryngopharyngeal symptoms, including sore throat and hoarseness. Furthermore, flexible LMA achieved better hemodynamic profile during intubation and less buckling during extubation.

Many studies have shown that the incidence of sore throat was higher after thyroid surgery when compared to general cases that did not involve neck surgery. This problem may be attributed to tracheal mucosal trauma, laryngeal edema, vocal cord hematoma or bilateral vocal cords palsy [12–14].

As a supraglottic device, LMA is positioned superior to the larynx and thus can cause less tracheal injury [15]. The use of LMA in general anesthesia could lower the incidence of postoperative sore throat than an ETT [16]. Although LMA may be dislocated during thyroidectomy due to neck hyperextension and surgical manipulation, the successful use of FLMA in head and neck surgeries [16, 17] and gained more and more popularity because it may protect the airway with less postoperative airway morbidity [18–20]. However, information about its use in thyroidectomy and the incidence of postoperative laryngopharyngeal discomfort is rare. Jung-Hee Ryu et al. [9] reported that an FLMA placed during thyroidectomy decreased the incidence and severity of postoperative laryngopharyngeal symptoms [21]. However, the reported incidence of sore throat in Jung-Hee Ryu’s study (97–100%) was significantly higher than that in other studies (68.4%), and the sample size was not large. Thereby we aimed to revalidate the hypothesis that FLMA was superior to ETT in reducing postoperative sore throat.

The causes for postoperative laryngopharyngeal discomfort were complicated. Several prospective clinical trials aimed to illustrate the risk factors for postoperative sore throat. Tracheal-tube size was considered to be a strong risk factor by different researchers [1, 6, 8]. The application of topical lignocaine gel to the ETT cuff may also affect the general incidence of sore throat, although its effect is controversial [8, 22]. In order to strictly control those potential confounding factors, a relatively smaller ETT was chosen and topical oxybuprocaine gel was applied to the ETT cuff in our study. Previous studies reported that higher cuff pressure of LMA might be associated with higher incidence of sore throat [23, 24], so the cuff pressure of flexible LMA was limited to 40 mmHg in our study to rule out the injury caused by overinflated cuff. Hisham AN. et al. suggested that extent of surgical procedure were also significant contributing factors affecting the postoperative recovery [8]. Therefore, we only recruited patients with thyroid cancer underdoing radical thyroidectomy, and all the surgeries were carried out by one surgeon group. Finally, analgesia methods may also have impacts on the laryngopharyngeal symptoms. A recent study indicated that high-dose intraoperative remifentanil infusion is associated with increased incidence of postoperative sore throat [25]. To minimizing such confounding effects, patients included in our study received standard intraoperative pain management strategy and no additional analgesics were given postoperatively.

The reasons why LMA is superior to ETT in reducing laryngopharyngeal discomfort may also be multifactorial. First, the movement of the ETT during positioning or traction on the trachea in thyroidectomy is an important attributor. This type of injury could be avoided with the use of flexible LMA. Second, the extent of surgical traction could be reduced because the mild leakage around LMA during manipulation may alert the surgeon of the excessive traction. Therefore, the operation may be more meticulous with an LMA airway. Less traction and gentler manipulation may result in less inflammation and edema in surgical area, and thereby less postoperative sore throat. However, this hypothesis needs further validation.

Our study was limited in the following aspects. First, the air seal of LMA was satisfactory in radial thyroidectomy in our study. However, the safety of LMA should be prudently evaluated if estimated surgery time is longer than 4 h. Second, since the anesthesia and operations were performed by experienced physicians, our conclusions might be less applicable in medical centers lacking specialized staff. Furthermore, the benefits for FLMA over ETT could be due to a more careful manipulation and less tissue injury from surgical team for the concern of FLMA displacement. Third, considering that hoarseness was assessed only by the change of voice, it could be more accurate if vocal chord evaluation or recurrent laryngeal nerve stimulation was used. Finally, recurrent laryngeal nerve injury is an obviously more serious complication of thyroid surgery than temporary postoperative sore throat and hoarseness, thus should be avoided by using an endotracheal tube with an electrode, which can help monitor of the function of recurrent laryngeal nerve. Therefore, LMA is not an appropriate choice in patients at high risk of nerve injury.

Our study provides clues for further research. First, considering the rapid developments of supraglottic device, the performance of other types of supraglottic device in thyroid surgeries can be investigated in the future. Second, the laryngopharyngeal symptoms after postoperative 48 h can also be further studied, since sore throat and hoarseness may last for 4 days or more.

## Conclusions

In conclusion, patients undergoing thyroid surgery with FLMA had less postoperative laryngopharyngeal symptoms, when compared with ETT. The use of FLMA also achieved less buckling during extubation and better hemodynamic profiles during intubation.

## Data Availability

The datasets generated and analyzed during the current study are available from the corresponding author on reasonable request.

## Abbreviations

ASA: American Society of Anesthesiology Physical Status Classification
BIS: Bispectral index
BMI: Body mass index
BW: Body weight
DBP: Diastolic blood pressure
EtCO_2_: Capnograpy
ETT: Endotracheal tube
FLMA: Flexible reinforced laryngeal mask airway
HR: Heart rate
LMA: Laryngeal mask airway
PUMCH: Peking Union Medical College Hospital
SBP: Systolic blood pressure
SpO_2_: Pulse oximetry
TCI: Target controlled infusions
VAS: Visual analog scale
